# Targeting Neural Endophenotypes of Eating Disorders with Non-invasive Brain Stimulation

**DOI:** 10.3389/fnins.2016.00030

**Published:** 2016-02-16

**Authors:** Katharine A. Dunlop, Blake Woodside, Jonathan Downar

**Affiliations:** ^1^Institute of Medical Sciences, University of TorontoToronto, ON, Canada; ^2^MRI-Guided rTMS Clinic, University Health NetworkToronto, ON, Canada; ^3^Department of Psychiatry, University Health NetworkToronto, ON, Canada; ^4^Department of Psychiatry, University of TorontoToronto, ON, Canada; ^5^Eating Disorders Program, University Health NetworkToronto, ON, Canada; ^6^Toronto Western Research Institute, University Health NetworkToronto, ON, Canada

**Keywords:** Eating Disorders (ED), RDoC, rTMS, tDCS, fMRI

## Abstract

The term “eating disorders” (ED) encompasses a wide variety of disordered eating and compensatory behaviors, and so the term is associated with considerable clinical and phenotypic heterogeneity. This heterogeneity makes optimizing treatment techniques difficult. One class of treatments is non-invasive brain stimulation (NIBS). NIBS, including repetitive transcranial magnetic stimulation (rTMS) and transcranial direct current stimulation (tDCS), are accessible forms of neuromodulation that alter the cortical excitability of a target brain region. It is crucial for NIBS to be successful that the target is well selected for the patient population in question. Targets may best be selected by stepping back from conventional DSM-5 diagnostic criteria to identify neural substrates of more basic phenotypes, including behavior related to rewards and punishment, cognitive control, and social processes. These phenotypic dimensions have been recently laid out by the Research Domain Criteria (RDoC) initiative. Consequently, this review is intended to identify potential dimensions as outlined by the RDoC and the underlying behavioral and neurobiological targets associated with ED. This review will also identify candidate targets for NIBS based on these dimensions and review the available literature on rTMS and tDCS in ED. This review systematically reviews abnormal neural circuitry in ED within the RDoC framework, and also systematically reviews the available literature investigating NIBS as a treatment for ED.

## Introduction

The term “eating disorders” (ED) encompasses a wide variety of disordered eating and compensatory behaviors that inappropriately alter the patient's body shape or weight, or the subjective experience of one's own body shape or weight. According to recent studies, the lifetime prevalence of EDs is 5.7% for females, and 1.2% in males (Golden et al., 2003; Hudson et al., 2007; Smink et al., 2014). The lifetime prevalence of the top three EDs according to the DSM-5 diagnostic criteria is 2.3, 1.7, and 0.8% for adolescent binge eating disorder (BED), anorexia nervosa (AN), and bulimia nervosa (BN), respectively (Golden et al., 2003; Hudson et al., 2007; Smink et al., 2014). BED is associated with recurrent episodes of binging, typically during negative affect (Leehr et al., 2015), and with the absence of inappropriate compensatory behaviors to avoid weight gain. Both AN and BN are associated with disturbances in the subjective experience of one's own body shape or weight; this phenotype is also known as body dysmorphia. BN is also defined by recurrent episodes of binge eating, with inappropriate compensatory behaviors to avoid weight gain; such behaviors include vomiting, excessive exercise, laxative misuse or diuretic misuse. In contrast, AN is defined by the persistent restriction of food, an intense fear of gaining weight, and a significantly low body weight for one's developmental stage. AN has two subtypes, restricting (ANR) and binge-eating/purging (ANBP), with the latter distinguished from the former by the presence of binges and/or purges.

Despite a low lifetime prevalence rate relative to other psychiatric disorders, EDs carry a significant burden of illness, both socially and individually. Treatment capacity in specialized ED programs is presently inadequate to meet demand (Hart et al., 2011), and for patients who do manage to access specialized programs, economic difficulties and high costs often hamper treatment adherence (Gatt et al., 2014). EDs are also associated with a high mortality rate; for one, approximately 10% of AN sufferers will die within 10 years of disease onset (Sullivan, 1995). According to a recent meta-analysis, the overall standard mortality ratio (SNR) for AN is 5.86, higher than schizophrenia (2.8), bipolar disorder (2.1), and major depression (1.6) (Arcelus et al., 2011). Conventional ED treatments, including pharmacotherapy, and in- and out-patient behavioral therapies, are associated with suboptimal recovery rates (~50% for AN; ~45% for BN; ~50–70% for BED), high relapse rates (ranging from 9 to 65%), and high chronicity (~20% will develop a chronic disorder; Olmsted et al., 2005; Mitchell et al., 2007; Shapiro et al., 2007; Carter et al., 2012; Hay et al., 2012; Hilbert et al., 2012; Amianto et al., 2015). ANBP, in particular, has the poorest prognosis of the eating disorders (Steinhausen and Weber, 2009). EDs are also highly co-morbid with other psychiatric disorders, such as major depression and obsessive-compulsive disorder, whose presence negatively impacts treatment outcomes (Godart et al., 2003; Crane et al., 2007; Mischoulon et al., 2011). Thus, new treatment approaches are urgently needed, especially for the substantial proportion of ED patients who are unresponsive to conventional treatment strategies.

Neuromodulation technologies are beginning to emerge as a promising new treatment option for treatment resistant ED patients. The potential usefulness of these techniques was recently illustrated in a pilot study using subgenual cingulate deep brain stimulation (DBS) to achieve symptomatic improvements in severe and treatment-refractory AN (Lipsman et al., 2013). Although potentially powerful, DBS remains for the moment a fairly invasive treatment, and is available only to small volumes of patients in specialist neurosurgical centers. A more accessible alternative is non-invasive brain stimulation (NIBS), including techniques such as repetitive transcranial magnetic stimulation (rTMS) and transcranial direct current stimulation (tDCS). rTMS uses rapid pulses of an electromagnetic field to elicit action potentials in the target area of cortex. tDCS uses a weaker intensity electrical stimulus, delivered by scalp electrodes, to modulate cortical excitability in the underlying regions. Both NIBS strategies attempt to alter the cortical excitability of a target brain region to normalize particular disorder-specific phenotypes. Cortical targets are typically selected based on abnormal structural or functional attributes in the disorder relative to healthy controls. Appropriate cortical targeting using NIBS is critical for optimal treatment efficacy (Fox et al., 2013). Therefore, a proper understanding of the neural substrates, as well as the cognitive and behavioral phenotypes accompanying these substrates, is crucial for optimizing future treatments.

Two major issues associated with NIBS as a treatment for ED are the tremendous heterogeneity in the cognitive and behavioral phenotypes of patients within this illness category, and the dynamic course of the illness, in which patients can switch from one ED diagnosis to another over time (Garfinkel et al., 1995; Keel and Mitchell, 1997; Lilenfeld et al., 1998; Sullivan et al., 1998; Strober et al., 2000; Bulik et al., 2005; Milos et al., 2005). This variability within a single diagnosis and this malleability of symptoms is a likely contributor to the limited clinical efficacy of NIBS (and in conventional treatments more generally) for ED.

Two possible solutions to address this heterogeneity are genomic methods, such as phenotypic linkage analyses, as well as neuroimaging methods. Such tools stratify patients on underlying behavioral, genetic, and neuropathological dimensions rather than self-reported symptoms alone. Therefore, these tools may be useful to identify the underlying behavioral and neuropathological endophenotypes related to more basic dimensions of behavior, independent of DSM-5 diagnoses. Such analyses and resulting endophenotypes can also be related to the behavioral and circuit-based dimensions of the recently described Research Domain Criteria (Insel et al., 2010) (RDoC). The RDoC is a recent strategy aimed at integrating basic neuroscientific knowledge with clinical diagnoses by first describing fundamental behaviors, described below, as dimensions. These dimensions are then used to describe the pathological behaviors of psychiatric disorders. By using the RDoC schema in combination with neuroimaging and phenotypic linkage methods, we may be able to identify sufficient stimulatory targets addressing specific phenotypes such as restrictive behavior or binging, regardless of DSM-5 diagnosis. For NIBS treatments, diagnostic systems must be capable of parsing this heterogeneity using endophenotypes so we may select the optimal stimulation target for a particular behavioral marker, or neural substrate.

Here, we will review NIBS as a treatment for the three most prevalent forms of ED: AN, BN, and BED. First, we will posit candidate dimensions as outlined by the RDoC and their underlying behavioral and neurobiological targets associated with ED as potential candidates for NIBS. Second, we will review the available literature on rTMS and tDCS as possible treatments for ED. Lastly, we will discuss the current limitations of the NIBS-ED field, and opportunities of future study and development.

## Going beyond the DSM-5 diagnosis: How can we maximize efficacy?

As discussed above, one of the major obstacles in ED diagnosis and treatment is the heterogeneity within each diagnostic category; conversely, comparisons of clinical and psychological features across patients suggest that there is significant overlap between ED diagnoses (Garfinkel et al., 1995; Lilenfeld et al., 1998; Sullivan et al., 1998; Strober et al., 2000). Compounding this problem is the evolution of the illness, such that patients may transition from one diagnostic category to another over time (Bulik et al., 2005). For example, it is estimated that approximately 50% of patients initially diagnosed with ANR will develop binge/purge behaviors, and approximately 30% of BN patients have a history of AN (Keel and Mitchell, 1997). In another study following DSM-IV-diagnosed AN and BN, only one third of subjects retained their original diagnosis after 30 months (Milos et al., 2005). To improve diagnostic consistency and treatment efficacy may require us to identify more stable, more granular, and more biologically based subgroups, or endophenotypes, within the ED population.

Some classification efforts have focused on a single DSM diagnosis. AN has been subdivided into 3 stable classes based on co-occurring symptoms: fat-phobic ANR, fat-phobic ANBP, and non-fat-phobic ANR (Wildes et al., 2013). BN has been subdivided based on personality attributes (affective-perfectionistic, impulsive and low-comorbid psychopathology clusters Wonderlich et al., 2005) and based on presenting symptoms (binging, purging, and bingeing-purging, Striegel-Moore et al., 2005).

A number of latent class (LCA) and latent profile analyses (LPA) have been performed on symptomatic and personality factors to stratify endophenotypes spanning AN and BN. One symptom-based LCA found optimal fitting for a 4-group classification. ANR, ANBP/BN, ANR without OCD, and BN with only vomiting as purging were the four groups identified (Keel et al., 2004). Another symptom-based LPA identified 4 ED classes: binging with multiple types of compensatory behavior; binging with only vomiting as compensatory behavior; binging without purging; and low/normal weight with excessive exercise (Eddy et al., 2009).

As evidenced above, there now exist a variety of different proposals for how best to subcategorize ED patients, within and across DSM-5 diagnoses. How, therefore, can we converge upon a system that offers maximum clinical usefulness? One potentially fruitful method would be to better characterize the heterogeneity among ED patients in biological terms, using techniques such as positron emission tomography (PET), electroencephalography (EEG) and magnetic resonance imaging (MRI) to identify distinct neurobiological substrates underlying the different subgroups within ED. Clinical endophenotypes could then be tied to neurobiological substrates, which could in turn serve as targets for individually- or phenotypically-tailored treatment strategies.

**Figure 1:**
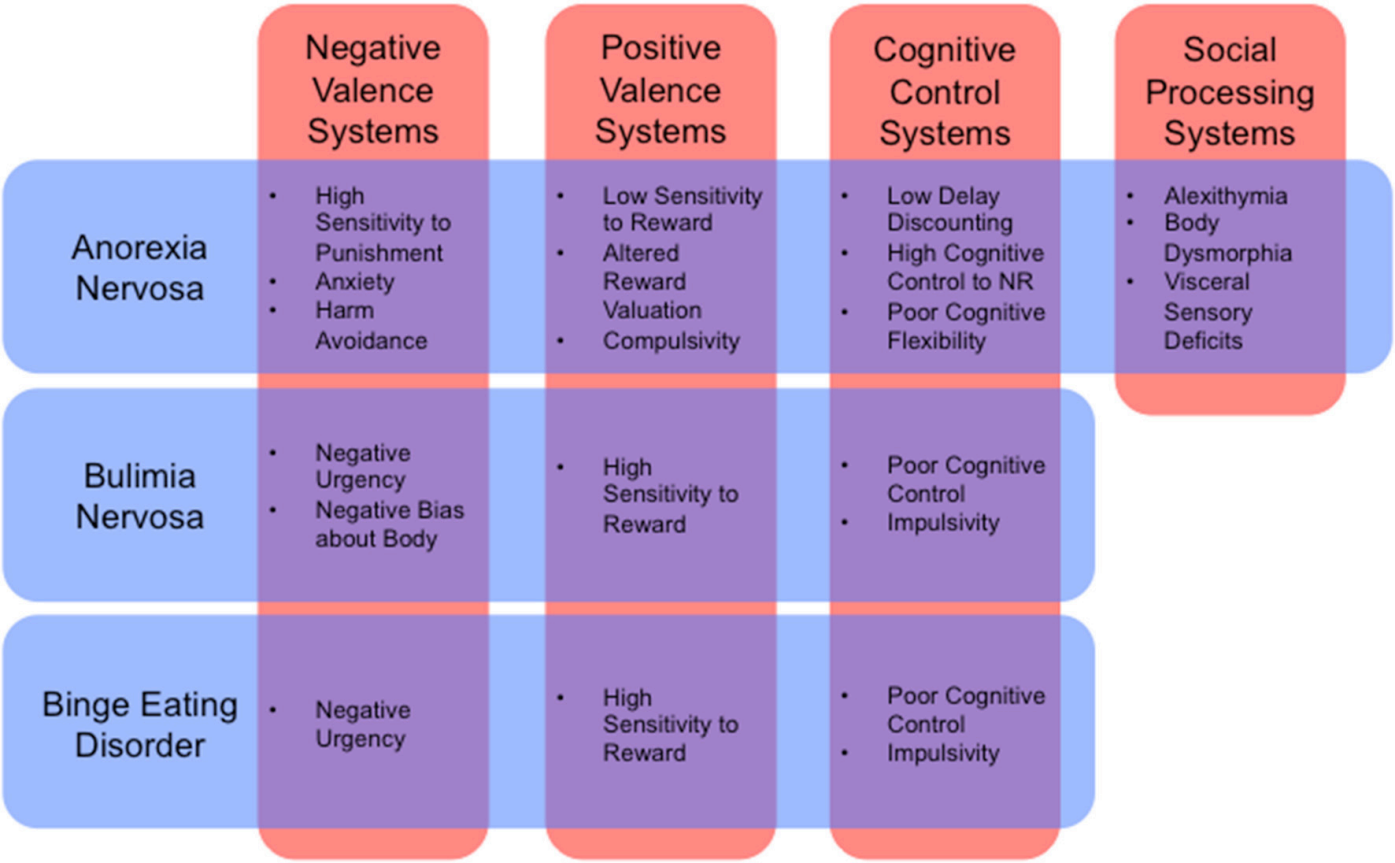
**Cognitive and behavioral phenotypes by RDoC dimension (Insel et al., 2010) for anorexia nervosa, bulimia nervosa, and binge eating disorder (Schebendach et al., 2007, 2013; Fernández-Aranda et al., 2008; Zastrow et al., 2009; Harrison et al., 2010; Klein et al., 2010; Miyake et al., 2010; Manwaring et al., 2011; Bohon and Stice, 2012; Hoffman et al., 2012; Steinglass et al., 2012; Chan et al., 2013; Giel et al., 2013; Strigo et al., 2013; Wu et al., 2013; Glashouwer et al., 2014; Kullmann et al., 2014; Mole et al., 2015; Berg et al., 2015; Racine et al., 2015; Tapajóz P de Sampaio et al., 2015)**. NR, Natural Rewards.

Such an approach would also allow us to describe illnesses in dimensional rather than categorical terms. For example, the influential RDoC framework (Insel et al., 2010) includes dimensional constructs such as positive valence, negative valence, cognitive systems, social processes, and arousal and regulatory systems (for a review of how RDoC dimensions relate to ED neurobiology, see Wildes and Marcus, 2015, Table 1). Many endophenotypes, previously identified as symptom clusters in the ED population, can be framed parsimoniously as the result of pathology affecting these dimensions, either singly or in combination (Figure 1). An “RDoC formulation” of our ED endophenotypes carries the advantage of pointing toward specific cognitive processes, neural pathways, neurotransmitter systems, molecular targets, or genes that might be targeted for therapeutic effect. For the purposes of this review, we will confine our discussion to potential novel uses of NIBS to target specific neural pathways that are associated with RDoC constructs, as they relate to specific endophenotypes within the ED population.

**Table 1:** **Overview of the 5 Research Domain Criteria domains as adapted from Insel et al. (2010) and Morris and Cuthbert (2012)**.

**RDoC Domain**	**Construct**
Negative valence systems	Active threat/Fear
	Potential threat/Anxiety
	Sustained threat
	Loss
	Frustrative nonreward
Positive valence systems	Approach motivation
	Responsiveness to reward
	Reward learning
	Habit
Cognitive systems	Attention
	Perception
	Working/Declarative memory
	Language
	Cognitive/Effortful control
Social processes	Imitation/Theory of mind
	Social dominance
	Facial expression identification
	Attachment/Separation
	Self-Representation
Arousal/Regulatory systems	Arousal
	Circadian rhythms
	Sleep and wakefulness

## RDoC domains as ED endophenotypes and NIBS targets

For the following section, a systematic review was completed using PubMed (NIH, http://www.ncbi.nlm.nih.gov/pubmed), with searches containing the following terms: first, clinical terms for the three ED diagnoses in this review (bulimia nervosa, anorexia nervosa, and binge eating disorder), and second, RDoC related terms as discussed in a recent review on RDoC cognitive systems (Morris and Cuthbert, 2012).

### Negative valence systems

Negative valence systems are activated in response to aversive stimuli, and include fear, anxiety and loss-related behaviors. In a recent meta-analysis investigating neural activations for negative and positive affect, negative valence was associated with greater activation in the amygdala and anterior insula (Lindquist et al., 2015). The lateral orbitofrontal cortex (OFC) is also associated with negative valence, particularly during the anticipation and receipt of punishment (Ursu et al., 2008).

A number of studies support the role of negative valence systems in ED, mainly in behaviors associated with negative affect, sensitivity to punishment, anxiety, harm avoidance, and response to the receipt of punishment (Figure 1). For example, behavioral measures of negative affect and negative urgency are the two most predictive features before a binge episode in both BED and BN (Bohon and Stice, 2012; Berg et al., 2015; Leehr et al., 2015; Racine et al., 2015). On functional neuroimaging, BN patient reported negative affect is related to neural responsivity during the anticipation of a food reward in both the striatum and insula (Bohon and Stice, 2012). This relation suggests that negative affect and food-reward are inappropriately coupled in this disorder. More generally, BN patients also have higher neural responses to negative body image descriptors (Miyake et al., 2010), in areas associated with the regulation and inhibition of fear and emotional processing circuits, including the dorsomedial prefrontal cortex (DMPFC) (Kühn et al., 2011; Åhs et al., 2015). These findings shed light on the role of negative attentional bias in the psychopathology of bulimic-type disorders.

Restrictive subtypes of ED also show hypersensitivity on measures related to negative valence systems. Behaviorally, exaggerated harm avoidance and sensitivity to punishment are typically associated with forms of AN (Harrison et al., 2010). Similarly, on fMRI, AN patients display increased neural activation in right anterior insula and DLPFC during pain anticipation, and exaggerated responses to punishment (pain and monetary losses) in the DLPFC, and the anterior, mid-, and motor cingulate (Bischoff-Grethe et al., 2013; Strigo et al., 2013; Bar et al., 2015). Cowdrey and colleagues also found an exaggerated response to an aversive taste and sight of food in the insula, striatum and ACC (Cowdrey et al., 2011). Trait-anxiety is also a common feature of AN, and is associated with the exaggerated activity of fear-related circuits to food and body-related cues. Regions of exaggerated response to symptom-provoking stimuli include the amygdala, hippocampus, insula, ACC, and medial PFC (Ellison et al., 1998; Frank et al., 2002, 2012b; Seeger et al., 2002; Uher et al., 2004; Friederich et al., 2010; Vocks et al., 2010). Finally, at the receptor level, PET imaging reveals increased striatal dopamine binding potential and altered cingulate serotonergic (increased 5-HT1A, but decreased 5-HT2A) binding potential is associated with harm avoidance in AN (Bailer et al., 2004, 2007; Frank et al., 2005).

#### Summary of potential negative valence targets

**Figure 2:**
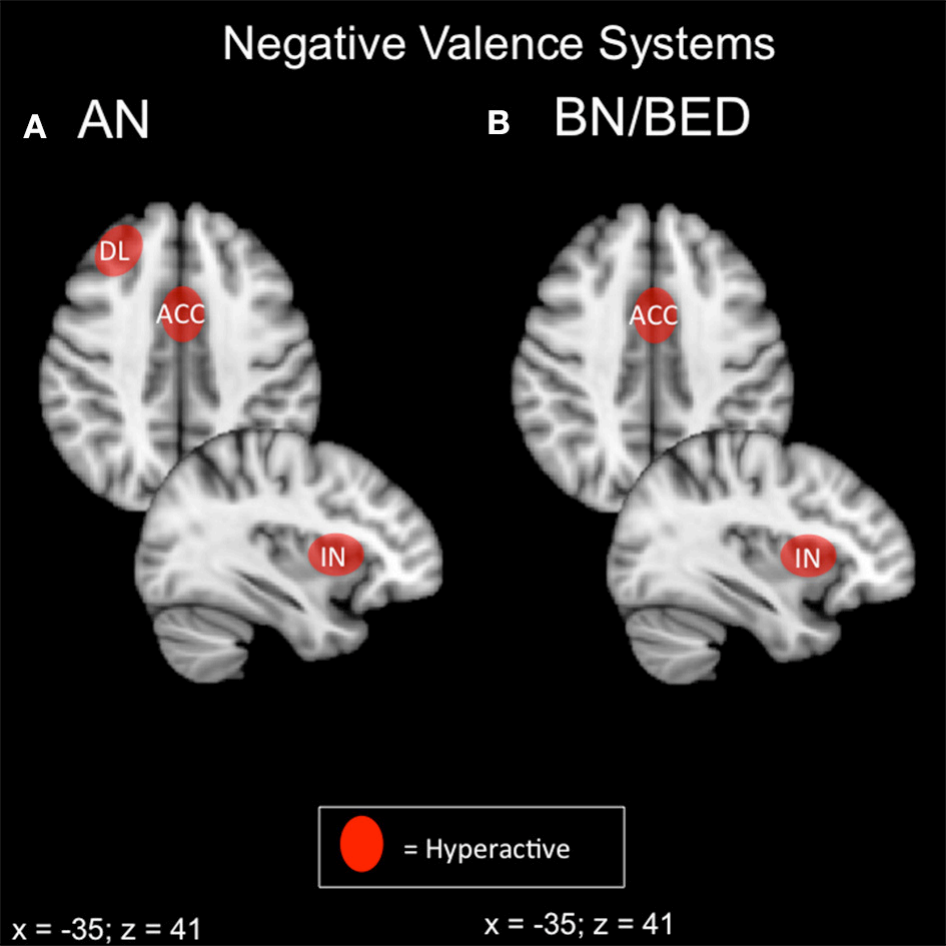
**Candidate NIBS targets that address abnormal phenotypes related to the RDoC negative valence dimension**. **(A)** Candidate negative valence NIBS targets for anorexia nervosa (AN) (Ellison et al., 1998; Frank et al., 2002, 2012b; Seeger et al., 2002; Uher et al., 2004; Friederich et al., 2010; Vocks et al., 2010; Cowdrey et al., 2011; Bischoff-Grethe et al., 2013; Strigo et al., 2013; Bär et al., 2015). The dorsolateral prefrontal cortex (DL) is abnormally hyperactive for pain anticipation and the receipt of punishment. The anterior cingulate cortex (ACC) is hyperactive for aversive food stimuli, the receipt of punishment, and anxiety. Finally, the anterior insula (IN) is abnormally hyperactive during anxiety and the anticipation of pain. **(B)** Candidate negative valence NIBS targets for bulimia nervosa (BN) and binge eating disorder (BED). The ACC is abnormally activated for negative words about the body (Miyake et al., 2010), while the insula is hyperactive during negative affect (Bohon and Stice, 2012).

Both bulimic and restrictive-type EDs display some form of negative valence abnormality on behavioral and neuroimaging modalities (Figure 2). In ED with a binging component, it appears that negative affect and food-reward responsivity are intimately coupled via the exaggerated response of the amygdala, insula and DMPFC. Restriction-related EDs display a similar pattern in the amygdala, right anterior insula, DLPFC and mPFC accompanying aspects of harm avoidance and receipt of punishment. Frontal regions, particularly the medial PFC and DMPFC, are thought to inhibit activity of the basolateral amygdala (BLA) (Cho et al., 2013; Felix-Ortiz et al., 2015).

Hyperactivation of the DMPFC, DLPFC, and anterior insula during negative valence paradigms has two possible interpretations. First, these areas may be inhibiting BLA activity, but insufficiently, in which case excitatory stimulation may be beneficial. Alternatively, these areas may actually be inappropriately driving BLA activity in a top-down fashion, in which case inhibitory stimulation would be preferable. A key study illustrated these opposite mechanisms in emotion regulation in healthy controls vs. MDD patients (Johnstone et al., 2007): during emotional reappraisal, limbic frontal regions suppressed amygdala activity in controls, but counterproductively *increased* amygdala activity in MDD.

For NIBS interventions, direct suppression of the amygdala is challenging due to its deep location; strategies aimed at damping negative valence systems will therefore likely target in prefrontal cortex and insula. Excitatory prefrontal NIBS has been recently shown to attenuate amygdala-dependent negative processing in healthy controls (Baeken et al., 2010; Guhn et al., 2014), and this strategy may be best in “bottom-up” pathology, where emotional reappraisal/self-regulation systems are underactive rather than pathologically hyperactive (i.e., in BN and BED). Conversely, where negative valence systems are driven by “top-down” pathology, and self-regulation is if anything excessive, inhibitory stimulation may be preferable. Inhibitory NIBS of the DMPFC and lateral OFC both show promise in obsessive-compulsive disorder (Mantovani et al., 2010; Nauczyciel and Drapier, 2012; Dunlop K. et al., 2015), and these strategies may be better suited to AN-R, particularly in cases with comorbid OCD.

### Positive valence systems

Positive valence systems encompass neural circuits related to motivation, reward seeking, and habit formation behaviors. According to a recent meta-analysis in healthy controls, positive stimuli are associated with activity in the VMPFC and ACC (Lindquist et al., 2015). All three major EDs, AN, BN, and BED, have been previously shown to be altered in this dimension (Figure 1).

From a behavioral perspective, AN patients show diminished sensitivity to conventional reward, as evident on psychometric measures (Harrison et al., 2010; Glashouwer et al., 2014) and delay discounting tasks (Steinglass et al., 2012). From a neurobiological perspective, ANR patients likewise display a blunted neural response to food reward in the insula and striatum (Wagner et al., 2008), decreased response to food images in the insula (Holsen et al., 2012; Oberndorfer et al., 2013b), and altered striatal activation during a reward-learning paradigm (Wagner et al., 2007). In a recent fMRI study of delay discounting in AN patients and healthy controls, AN patients had a marked preference for delayed rewards, associated with lower activation in the striatum and dorsal ACC during decision-making; these behavioral and neural abnormalities normalized to control levels after treatment (Decker et al., 2014). However, another study found that weight restoration did not affect choice behavior on a delay discounting task (Ritschel et al., 2015), suggesting that a preference for delayed over immediate rewards may be an endophenotypic feature in low-BMI individuals. In either case, the identified striatal and prefrontal regions are all involved in the motivational aspect of reward and food-reward processing.

There is also evidence that reward evaluation is altered in AN, in which secondary (contextual) rewards such as exercise and dietary restriction carry higher reward value relative to food or other primary rewards (Schebendach et al., 2007; Klein et al., 2010). The so-called “reward contamination theory” of AN posits a pathological re-configuration of the patient's reward system through stress-induced activation of the mesolimbic dopamine system, via ventral tegmental area opioid receptors. In this framework, AN behaviors essentially represent a maladaptive, but well-entrenched type of habit-formation (Keating et al., 2012; Walsh, 2013).

The findings that support this theory suggest that there is altered motivational salience for disease-related stimuli. For example, AN patients tend to rate physical exercise as “pleasant,” more so than food (Giel et al., 2013). In fact, food-reward in AN activates a weight-gain fear response (i.e., negative valence systems) in the amygdala and extrastriate body rather than positive valence systems from the striatum, orbitofrontal cortex, and ACC (Vocks et al., 2011). The DLPFC is also hyperactive in response to images of food and the anticipation of reward, suggesting the presence of enhanced cognitive control over food cues and reward (Ehrlich et al., 2015; Sanders et al., 2015). ANR patients also have a high prevalence of comorbid OCD (Torresan et al., 2013). The level of compulsivity predicts the reactivity of the superior frontal gyrus, ACC and striatum and deactivation of the PFC to images of high-calorie foods (Rothemund et al., 2011), and lowered right DLPFC activity is seen in response to obsessive-compulsive symptom provocation in AN (Suda et al., 2014). Thus, hypofunctioning of primary reward systems (and potentially, hyperfunctioning of secondary/contextual reward systems) may be important target processes in ANR.

In contrast disorders in the BN/BED spectrum are often associated with elevated primary reward valuation and reward sensitivity. These are typically associated with a higher willingness to work for a food reward (Schebendach et al., 2013), as well as higher impulsivity (Manwaring et al., 2011; Chan et al., 2013; Mole et al., 2015) At the neural level, BN and BED patients show increased activity for reward receipt in areas including the medial OFC, ventral striatum and insula (Schienle et al., 2009; Frank et al., 2011, 2012a; Radeloff et al., 2012; Weygandt et al., 2012; Oberndorfer et al., 2013a). BED patients display hyperactivations in the ventral striatum and inferior frontal gyrus during reward anticipation, and reduced medial PFC activity during a monetary incentive delay task (Balodis et al., 2014, 2013a). On PET imaging, areas like the insula, PFC and ventral striatum, associated with reward-motivation and food-reward processing, have altered serotonergic and dopaminergic binding in BN (Broft et al., 2012; Galusca et al., 2014). An important associated feature may also be deficient behavioral self-regulation and impulsivity. BN patients also show reduced activation in anticipation of a food reward is seen in ACC and right anterior insula; lower ACC activity predicts how much the patient will overeat (Frank et al., 2006; Bohon and Stice, 2011). Parallels have been drawn between the neural substrates of BN/BED and addiction, due to the similar alterations to motivation and reward-related circuitry on fMRI and task-based paradigms between the two psychopathologies (Filbey et al., 2012).

#### Summary of potential positive valence targets

**Figure 3:**
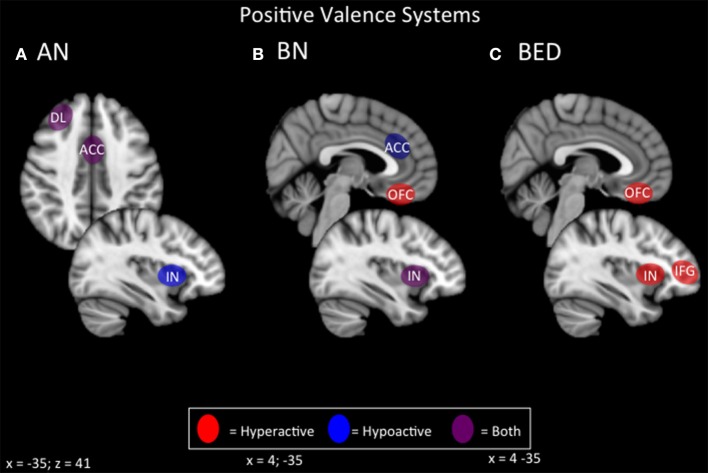
**Candidate NIBS targets that address abnormal phenotypes related to the RDoC positive valence dimension**. **(A)** Candidate positive valence NIBS targets for anorexia nervosa (AN) (Wagner et al., 2007, 2008; Rothemund et al., 2011; Vocks et al., 2011; Holsen et al., 2012; Oberndorfer et al., 2013b; Torresan et al., 2013; Decker et al., 2014; Suda et al., 2014; Ehrlich et al., 2015; Sanders et al., 2015). The dorsolateral prefrontal cortex (DL) is both hyperactive when the participant views images of food, but hypoactive during symptom, particularly OCD-related, provocation. The anterior cingulate cortex (ACC) is also differentially activated; it is hyperactive when the participant views images of food, but hypoactive when the participant delays a reward. Also, the insula (IN) is hypoactive when the participant views images of food. **(B)** Candidate positive valence NIBS targets for bulimia nervosa (BN) (Frank et al., 2006, 2011; Bohon and Stice, 2011; Broft et al., 2012; Radeloff et al., 2012; Weygandt et al., 2012; Oberndorfer et al., 2013a; Galusca et al., 2014). The ACC is hypoactive during reward anticipation, and this hypoactivity predicts later overeating. The orbitofrontal cortex (OFC) is hyperactive during the receipt of a reward. The IN is both hyperactive during the receipt of a reward, but hypoactive during reward anticipation. **(C)** Candidate positive valence NIBS targets for binge eating disorder (BED) (Schienle et al., 2009; Frank et al., 2012a; Weygandt et al., 2012; Balodis et al., 2013a, 2014). Both the OFC and the IN are abnormally hyperactive during the receipt of a reward, while the inferior frontal gyrus (IFG) is hyperactive during reward anticipation.

In terms of positive valence systems, it appears that both restrictive and binging phenotypes of ED display alterations in incentive salience that is potentially modulated by the opioid system (Keating et al., 2012; Giuliano and Cottone, 2015; Figure 3). In the case of ANR, conventional primary rewards appear to be devalued in favor of pathological secondary or contextual rewards, such as starvation and excessive exercise. A broader preference for long-term/contextual over immediate primary rewards is also apparent in choice behavior during delay discounting. Neurally, the primary reward systems of the ventral striatum and ventromedial prefrontal cortex appear hypoactive, while contextual or secondary reward systems operating through lateral orbitofrontal and lateral temporal regions appear hyperactive. Hyperactivity in lateral orbitofrontal pathways is also strongly associated with OCD, and with compulsivity in general (Ahmari et al., 2013; Beucke et al., 2013). This finding would be consistent with the broader phenotype of ANR. Neurally-based strategies in ANR might therefore include enhancing primary reward value via medial prefrontal-striatal pathways, or attenuating secondary reward value via lateral prefrontal-striatal pathways. For instances where conventional rewards are less valued than maladaptive ones (restrictive, fat-phobic ED), inhibitory NIBS over lateral networks for maladaptive secondary rewards, and excitatory NIBS over medial networks for conventional rewards, may be a possible therapeutic protocol to realign incentive-salience mechanisms to normal, adaptive functioning.

In the case of binge/purge-related EDs, repeated exposures to the transient reward value of food intake (or the transient anti-anxiety effect of purging) would cause these behaviors to acquire pathologically high incentive value (especially in the presence of negative urgency), via neural mechanisms that parallel those of addiction. Effective strategies would therefore parallel those for substance addiction: enhancing cognitive/impulse control over urges to binge and purge, or suppressing urge intensity.

NIBS strategies for enhancing cognitive control involve excitatory stimulation of the nodes of the salience network, including the DLPFC, dACC, and insula (Dunlop et al., accepted). Each of these targets have demonstrated efficacy in substance dependence (Mishra et al., 2010; De Ridder et al., 2011; Meng et al., 2014), with effects apparently mediated by enhanced control rather than reduced urge. Recently, excitatory rTMS over the dACC has been reported to reduce symptoms in treatment-resistant binge/purge ED, via enhanced integrity of frontostriatal circuits in the salience network (Dunlop J. et al., 2015).

NIBS may also be capable of suppressing urge, by targeting frontopolar and ventromedial sites. In one preclinical rTMS study, substance use disorder patients underwent inhibitory rTMS over the ventral frontal pole during a task evoked a cue-related craving response. A single session of inhibitory rTMS reduced the severity of craving in this group relative to sham, and stimulation proved capable of engaging core reward nodes in the ventral striatum, as well as the associated ventromedial prefrontal regions (Hanlon et al., 2013, 2015). Urge suppression via inhibitory ventromedial prefrontal stimulation has yet to be attempted in ED, but would be a reasonable strategy to complement excitatory salience-network stimulation in binge/purge-related ED populations.

### Cognitive systems

The cognitive systems dimension refers to processes responsible for cognitive processing, including attention, perception, memory, language, and cognitive control. In healthy control studies, these behaviors are associated with activity in the DMPFC, DLPFC, and anterior insula (Albares et al., 2014; Cho et al., 2014; Luo et al., 2014; Reineberg et al., 2015). These networks tend to be associated with the central executive and salience resting-state networks (Reineberg et al., 2015), responsible for response selection and inhibition.

Abnormal cognitive control mechanisms are evident in most ED populations (Figure 1). On the one hand, BN and BED-type diagnoses tend to display reduced capacity for impulse and cognitive control. This is particularly evident for disease-relevant stimuli (Wu et al., 2013), but is also apparent for positive and negative emotional valence images (Tapajóz P de Sampaio et al., 2015), suggesting a broader endophenotype of deficient cognitive and behavioral control. In fact, binge episodes are partially defined by the individual's loss of control during eating, and impulse control disorders (ICD) are common comorbidities (Fernández-Aranda et al., 2008). Purging behaviors are also associated with higher levels of impulsivity, and different forms of purging may represent separate manifestations of compulsivity and impulsivity (Hoffman et al., 2012).

On the other hand, restrictive-type EDs tend to show a different profile of cognitive control abnormalities. Cognitive control capacity may appear above normal levels in certain domains, such as temporal discounting (Steinglass et al., 2012). However, cognitive control may be abnormal in certain specific domains related to the illness; for example, for negative valence images (Tapajóz P de Sampaio et al., 2015), food stimuli (Oberndorfer et al., 2013b; Sanders et al., 2015), or body-image-related stimuli (Lee et al., 2014). AN patients also have altered cognitive control depending on the reward valence of the object, as the impulse control networks are overly activated for physical exercise relative to food images in a go/no-go task (Kullmann et al., 2014). AN patients also show a reduced ability to switch to an optimal decision-making strategy, called cognitive flexibility (Zastrow et al., 2009).

From a neural perspective, impulsive-type deficits on response control tasks are related to lower frontostriatal activations. BED patients show reduced activity in the inferior frontal gyrus, ventromedial PFC and insula during the Stroop task, and this diminished activity is associated with poor dietary restraint (Balodis et al., 2013b). BED prefrontal hypoactivity has also been correlated with psychometric measures of attentional impulsiveness and a disease-relevant go/no-go task (Hege et al., 2014). BN patients show hypoactivity in frontostriatal circuitry during cognitive control tasks like the Simon Spatial Incompatibility task; affected areas include the inferior frontal gyrus, striatum, ACC, OFC, DLPFC, and middle frontal gyrus (Marsh et al., 2009, 2011; Celone et al., 2011). On the go/no-go task, adolescent BN and ANBP patients display hyperactivations in the ACC and right DLPFC, albeit without impaired task performance relative to controls (Lock et al., 2011).

AN patients also show hypoactivity in frontostriatal circuits from the medial PFC on a response inhibition task related to cognitive control deficits (Oberndorfer et al., 2011; Wierenga et al., 2014), but hyperconnectivity to a response inhibition task that used exercise-related stimuli as its cue (Kullmann et al., 2014). Additionally, AN patients also display poorer performance on cognitive flexibility tasks, and this performance is reflected by lower activity in frontostriatal circuits through the thalamus, ventral striatum, ACC, middle frontal gyrus, and ventrolateral PFC (Zastrow et al., 2009; Sato et al., 2013; Garrett et al., 2014; Wildes et al., 2014; Lao-Kaim et al., 2015). On resting-state fMRI, higher thalamo-cortical functional connectivity through the DLPFC and anterior PFC is associated with poorer performance on the Stroop task and working memory (Biezonski et al., 2015). Thus, domain-specific abnormalities of cognitive control are evident at both the behavioral and the neural level in AN.

#### Summary of potential cognitive control targets

**Figure 4:**
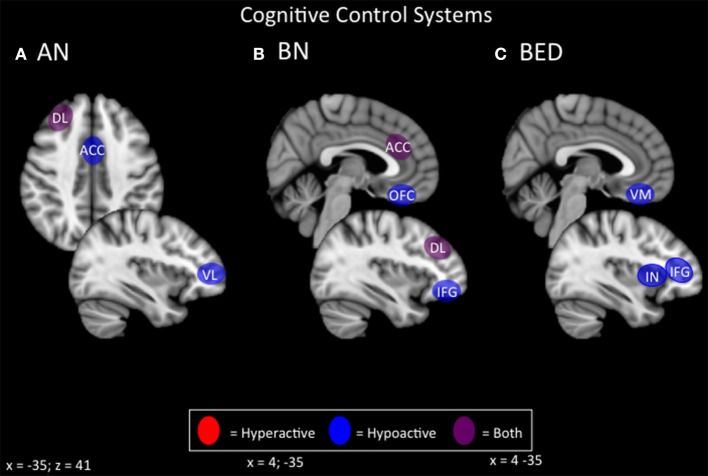
**Candidate NIBS targets that address abnormal phenotypes related to the RDoC cognitive control dimension**. **(A)** Candidate cognitive control NIBS targets for anorexia nervosa (AN) (Oberndorfer et al., 2011; Sato et al., 2013; Garrett et al., 2014; Wierenga et al., 2014; Wildes et al., 2014; Biezonski et al., 2015; Lao-Kaim et al., 2015). The dorsolateral prefrontal cortex (DL) is both hyperactive during interference control tasks (such as the Stroop task), and for working memory, but hypoactive during cognitive flexibility and set-shifting tasks. The anterior cingulate cortex (ACC) is hypoactive during response inhibition tasks, while the ventrolateral prefrontal cortex (VL) is hypoactive during cognitive flexibility tasks. **(B)** Candidate cognitive control NIBS targets for bulimia nervosa (BN) (Marsh et al., 2009; Rossi and Hallett, 2009; Celone et al., 2011; Lock et al., 2011). Both the ACC and the DL are hyperactive during response inhibition tasks, but hypoactive during interference control tasks, while both the orbitofrontal cortex (OFC) and inferior frontal gyrus (IFG) are hypoactive during inference control tasks. **(C)** Candidate cognitive control NIBS targets for binge eating disorder (BED) (Balodis et al., 2013b; Hege et al., 2014). The ventromedial prefrontal cortex (VM), insula (IN), and IFG are abnormally hypoactive during interference control, poor dietary restraint, impulsivity, and response inhibition.

Both restricting- and binge/purge-type EDs show deficits on tasks related to cognitive control, including behavioral inhibition, working memory, selective attention, and cognitive flexibility (Figures 1, 4). Generally, BED displays poorer response inhibition and lower activity in the inferior frontal gyrus and ventromedial PFC, both of which are accessible via excitatory forms of NIBS. BN and ANBP display lower activity in the inferior frontal gyrus, ACC, OFC, and DLPFC; all but the OFC are easily accessible for excitatory NIBS. As noted earlier, excitatory NIBS of salience-network nodes in DLPFC, DMPFC, and anterior insula appears to improve cognitive control and impulsivity even in healthy controls (Cho et al., 2014, 2010; Meng et al., 2014). Enhanced cognitive control, via improved frontostriatal connectivity through these salience-network nodes, may mediate recently reported improvements in binge and purge behaviors with excitatory DMPFC-rTMS (Dunlop J. et al., 2015). Similar effects via similar mechanisms should be expected for excitatory rTMS targeting DLPFC and anterior insula.

For AN, neural correlates of cognitive control show considerable variability depending on the task and valence of stimuli. On the one hand, AN patients in some studies show broad deficits of cognitive control and flexibility, and hypoactivity of the frontostriatal circuitry, during many tasks related to cognitive control; hence, excitatory NIBS might be beneficial if combined with cognitive tasks during stimulation. On the other hand, patients sometimes show the reverse pattern of hyperconnectivity and excessive cognitive control/compulsivity in these same circuits, within illness-specific domains; excitatory stimulation may therefore be unhelpful, or could potentially exacerbate illness. In keeping with this concern, high-frequency DMPFC-rTMS was recently reported to exert a paradoxical *inhibitory* effect on frontostriatal connectivity in a subpopulation of ED patients with high baseline connectivity; these patients showed symptomatic worsening rather than improvement (Dunlop J. et al., 2015). Thus, targeting cognitive control in AN-R may require a more nuanced approach than is the case for binge-purge symptoms.

### Social processing systems

Social processing systems refer to circuits involved in social communication, and the perception and understanding of oneself and others. Targets identified in healthy controls include the insula, responsible for interoception (Craig, 2002); the temporoparietral junction, for theory of mind-related processing (Saxe and Kanwisher, 2003); and higher-order visual processing regions, for processing one's own and others' faces (Hummel et al., 2013).

This dimension has received less attention in the ED literature relative to positive/negative valence systems and cognitive control (Figure 1). However, it may have relevance in AN patients, who show higher levels of alexithymia, deficits in visceral sensory perception or “interoception” (Craig, 2002; Strigo et al., 2013), and distorted perceptions of body shapes (Suchan et al., 2013). AN patients with higher levels of alexithymia show lower ACC, PCC, and right temporoparietal junction (TPJ) activation during social decision-making tasks (Miyake et al., 2009, 2012; McAdams and Krawczyk, 2011). More specifically, ANR patients display altered anterior and dorsal mid-insula activations based on the modality of interoception they are attending to (Kerr et al., 2015). On resting-state fMRI, AN patients also display increased functional connectivity from the anterior insula to the default mode network associated with self-reported problems with interoceptive awareness, suggesting a heightened level of cognitive control toward interoceptive processes (Boehm et al., 2014). AN patients also have altered neural responses to visually-presented body shapes, particularly in areas associated with visual processing and reward: the ventral striatum, extrastriate body area (EBA), DLPFC, parietal regions, medial PFC, and fusiform gyrus (Cowdrey et al., 2012; Spangler and Allen, 2012; Castellini et al., 2013; Fladung et al., 2013; Suchan et al., 2013; Suda et al., 2013; Fonville et al., 2014). Finally, two recent studies have also identified areas of abnormal activation in response to benevolent and malevolent social relationships. During benevolent social relations, AN patients tend to display reductions in DMPFC, possibility related to lowered reward valence for social reward and interaction (McAdams et al., 2015; Via et al., 2015).

**Figure 5:**
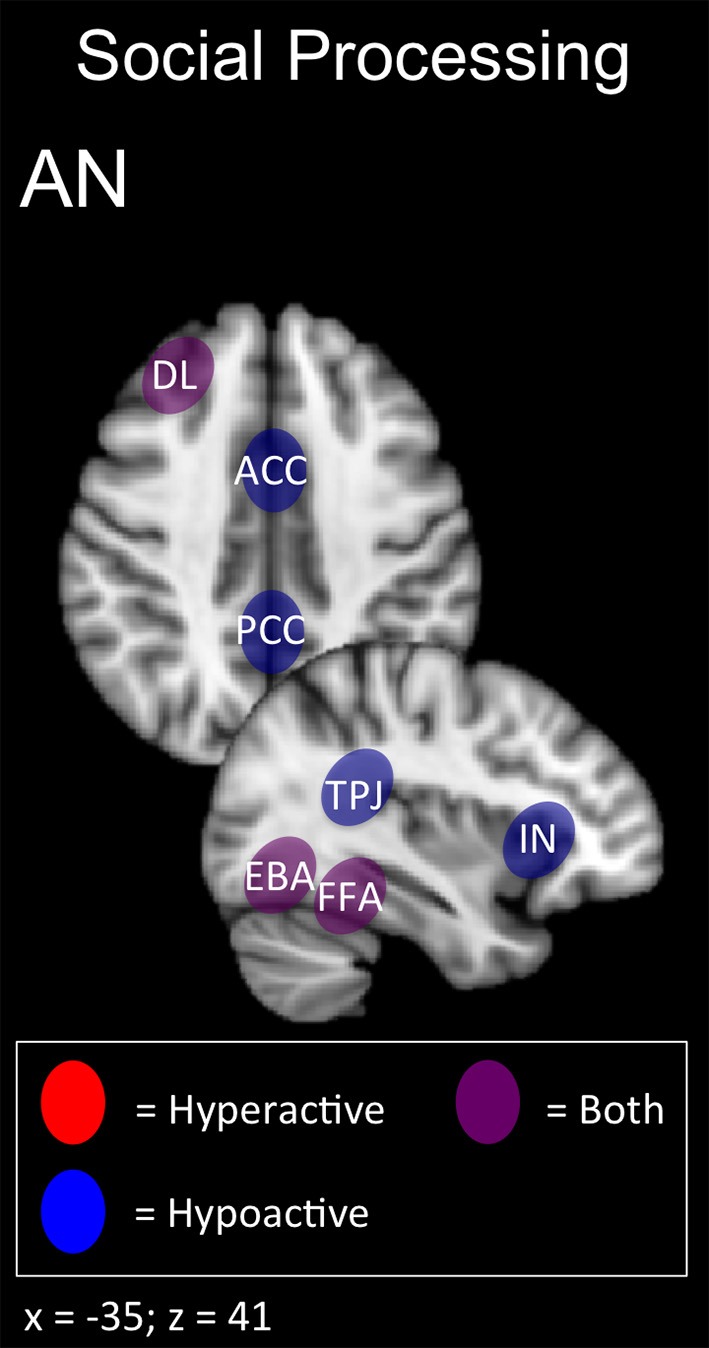
**Candidate NIBS targets that address abnormal phenotypes related to the RDoC social processing dimension in anorexia nervosa (AN) (Cowdrey et al., 2012; Miyake et al., 2012; Spangler and Allen, 2012; Castellini et al., 2013; Fladung et al., 2013; Suda et al., 2013; Boehm et al., 2014; Fonville et al., 2014; Kerr et al., 2015; McAdams et al., 2015; Via et al., 2015)**. The anterior cingulate cortex (ACC), posterior cingulate cortex (PCC), and temporoparietal junction (TPJ) are abnormally hypoactive during deficits in social decision-making and alexithymia, while low insula (IN) activity is related to deficits in interoceptive awareness. The dorsolateral prefrontal cortex (DL) is both abnormally hyperactive when the participant views oversized images of themselves, but hypoactive when viewing images depicting body-checking behavior. The fusiform face area (FFA) is both abnormally hyperactive when the participant views highly emotional facial expressions, but hypoactive when viewing distorted body shapes and images depicting body-checking behavior. The extrastriate body area (EBA) is both abnormally hyperactive when the participant views images of their own body, but hypoactive when those images are distorted.

In summary, AN patients may show deficits across multiple domains related to self-perception (alexithymia, interoception, and body shape perception) and social function (interpersonal interaction, theory of mind; Figures 1, 5). The latter function has been successfully enhanced with excitatory DMPFC-rTMS in autism-spectrum disorder (Enticott et al., 2011, 2014). During social interactions, AN patients likewise tend to display DMPFC hypoactivity during social interaction, and so excitatory stimulation over this region may worth exploring. For self-perception, relevant targets include anterior insula (alexithymia), posterior insula (interoception), TPJ and EBA (social cue perception, body shape perception). NIBS has successfully targeted each of these regions in other applications (Ciampi de Andrade et al., 2012; Dinur-Klein et al., 2014; Donaldson et al., 2015). Excitatory stimulation of the insula and TPJ may be worth exploring for alexithymia and deficits in interoception. Conversely, inhibitory stimulation of the TPJ and EBA may be worth exploring for aberrant self- and body perception.

## NIBS techniques as therapeutic interventions in ED

For the following section, a systematic review was completed using PubMed (NIH, http://www.ncbi.nlm.nih.gov/pubmed), with searches containing the following terms: first, clinical terms for the three ED diagnoses in this review and related phenotypes (BN, AN, BED, binging, purging, excessive exercise), and second, NIBS related terms (rTMS, TMS, tDCS).

### NIBS overview: rTMS and tDCS

rTMS applies powerful, focused magnetic field pulses over the scalp to elicit action potentials in the underlying region of cortex. Typically, treatment sessions occur once daily, for a total of 20–30 daily sessions (Carpenter et al., 2012; Solvason et al., 2014). rTMS mechanisms are thought to involve synaptic plasticity via long-term potentiation or depression, with the direction of effect dependent on the stimulation intensity, duration, and pattern (Pascual-Leone et al., 1998; Maeda et al., 2000). Higher frequency stimulation (5–20 Hz) is usually considered to be excitatory, while low frequency (< 1 Hz) stimulation is considered inhibitory (Pascual-Leone et al., 1994; Chen et al., 1997). More recently, however, considerable heterogeneity on electrophysiological, neuroimaging, and clinical measures has been found for most if not all patterns of rTMS (Maeda et al., 2000; Eldaief et al., 2011; Dunlop J. et al., 2015; Dunlop K. et al., 2015; Nettekoven et al., 2015).

tDCS, on the other hand, uses a constant, low amplitude current to modulate cortical excitability, rather than eliciting action potentials directly. As with rTMS, sessions typically occur daily, for a total of 10–30 sessions (Meron et al., 2015). While the mechanisms of tDCS are still debated, it is likely that modulated cortical excitability also elicits subtle effects on synaptic plasticity via long-term potentiation and depression (Brunoni et al., 2012). Anodal stimulation is considered excitatory, and cathodal stimulation inhibitory. However, as with rTMS, both types of tDCS display considerable inter-individual variability in their effects (Wiethoff et al., 2014). Newer variants such as transcranial alternating current stimulation (tACS), may exert more consistent, frequency-specific effects (Voss et al., 2014); however, their therapeutic potential is poorly understood at present.

### NIBS as a treatment for BED and food craving

To date, the majority of published NIBS-ED studies have focused on female patients with abnormally high food craving or urge to eat, as opposed to a specific formal DSM-5 diagnosis (Tables 2, 3; McClelland et al., 2013; Grall-Bronnec and Sauvaget, 2014; Val-Laillet et al., 2015). These preclinical studies typically involve a single session of stimulation, with subjectively rated cue-induced craving as the primary outcome. With rTMS, two studies reported contradictory results for 10 Hz stimulation of the left DLPFC rTMS: one study (*n* = 28) found decreased craving after active vs. sham stimulation (Uher et al., 2005), while the other (*n* = 10) found that active stimulation was no better than sham in terms of cue-induced craving control (Barth et al., 2011). The studies differed in stimulation parameters, however, and enrolled only healthy participants who self-reported having strong food cravings, but did not carry a formal ED diagnosis. Hence, it may be difficult to extrapolate these findings to the effects of a full therapeutic course of 20–30 sessions in patients with pathological deficits of self-control and a formal ED diagnosis.

**Table 2:** **Overview of the available ED-rTMS literature**.

**Study**	**Subjects**	**Study design**	**Stimulation Site**	**rTMS Technique**	**Duty cycle (on/off s)**	**Total pulses/session**	**Stimulation Intensity**	**Number of sessions**	**Primary outcome**	**Findings**
**A**										
Uher et al., 2005	*n* =28, F, FC	RCT	L-DLPFC, 5cm rule	10 Hz	5.55 s	1000	110% MT	1	Cue-induced urge to eat	Food craving increased after sham stimulation
Barth et al., 2011	*n* = 10, F, FC	RCT	L-PFC	10 Hz	10.20 s	3000	100% RMT	1	Inhibition of cue-induced cravings	rTMS no better than sham
**B**										
Van den Eynde et al., 2013	*n* = 10, AN	RCT	L-DLPFC, 5 cm rule	10 Hz rTMS	5.55 s	1000	110% MT	1	EDE-Q	Reduced levels of feeling full, fat, anxiety
**C**										
Van den Eynde et al., 2010	*n* = 38, bulimic-type ED	RCT	L-DLPFC, 5 cm rule	10 Hz	5.55 s	1000	110% MT	1	Binge-eating episodes, urge to eat	Decreased urge to eat and binges 24 h post-stimulation
Claudino et al., 2011	*n* = 22, bulimic-type ED	RCT	L-DLPFC	10 Hz	5.55 s	1000	110% MT	1	Salivary cortisol concentration	Lower cortisol post-rTMS compared to sham
Walpoth et al., 2008	*n* = 16, BN	RCT	L-DLPFC	20 Hz	10.60 s	2000	120% MT	15 sessions, daily	Binge/purge status	No significant difference between active and sham-rTMS
Hausmann et al., 2004	*n* = 1, MDD-BN	Case report	L-DLPFC	20 Hz	10.60 s	2000	80% MT	10 sessions, daily	Binge/purge Diary	Remission of binge/purges, HDRS response
Downar et al., 2012	*n* = 1, MDD-BN	Case report	B-DMPFC	10 Hz	5.10 s	6000	120% RMT	20 sessions, daily	Binge/purge status	Remission of binge/purges, HDRS response
Dunlop J. et al., 2015	*n* = 28, bulimic-type ED	Open-label	B-DMPFC	10 Hz	5.10 s	6000	120% RMT	20–30 sessions, daily	Binge/purge status	16 of 28 achieved >50% reduction of binges and purges 4-weeks post-rTMS

*A, rTMS studies related to food addiction and urges to eat; B and C, refer to rTMS studies related to AN and BN, respectively*.

*AN, anorexia nervosa; B-DMPFC, bilateral dorsomedial prefrontal cortex; BN, bulimia nervosa; ED, eating disorder; EDE-Q, eating disorder examination questionnaire; F, female; FC, food craving; HDRS, Hamilton Depression Rating Scale; L-DLPFC, left dorsolateral prefrontal cortex; L-PFC, left prefrontal cortex; MT, motor threshold; RCT, randomized control trial; RMT, resting motor threshold; rTMS, repetitive transcranial magnetic stimulation*.

**Table 3:** **Overview of the available ED-tDCS literature**.

**Study**	**Subjects**	**Study Design**	**Anodal Site**	**Cathodal Site**	**Stimulation Intensity**	**Number of sessions**	**Primary Outcome**	**Findings**
**A**								
Fregni et al., 2008	*n* = 23, HC with urges to eat	Sham-controlled, crossover	R-DLPFC	L-DLPFC	2 mA, 20 min	1	Craving VAS, food consumption	Reduced craving in active tDCS, less consumption
Goldman et al., 2011	n = 19, HC with urges to eat	Sham-controlled, crossover	R-DLPFC	L-DLPFC	2 mA, 20 min	1	Craving VAS, Resist food	Reduced craving, increased ability to resist food
Kekic et al., 2014	*n* = 20, HC with urges to eat	Sham-controlled, crossover	R-DLPFC	L-DLPFC	2 mA, 20 min	1	Craving VAS	Reduced craving for sweet foods
Lapenta et al., 2014	*n* = 9, HC with urges to eat	Sham-controlled, crossover	R-DLPFC	L-DLPFC	2 mA, 20 min	1	Cue-induced food craving	Reduced food intake
**B**								
Khedr et al., 2014	*n* = 7, AN	Open-Label	L-DLPFC	N/A	2 mA, 25 min	10, daily	EDI and EAT	Significant effect of time on EAT and EDI

*A, tDCS studies related to food addiction and urges to eat; B, tDCS studies related to AN*.

*AN, anorexia nervosa; HC, healthy controls; EAT, eating attitudes test; EDI, eating disorder inventory; L-DLPFC, left dorsolateral prefrontal cortex; R-DLPFC, right dorsolateral prefrontal cortex; tDCS, transcranial direct current stimulation; VAS, visual analog scale*.

There is also a growing body of literature investigating DLPFC-tDCS as a method to reduce craving and food intake. In four published studies recruiting individuals with strong food cravings, a single session of anodal right DLPFC/cathodal left DLPFC tDCS was able to reduce cue-induced craving, reduce food intake, and improve the participants' ability to resist food relative to sham-tDCS (Fregni et al., 2008; Goldman et al., 2011; Kekic et al., 2014; Lapenta et al., 2014). Future work involving tDCS should employ multiple sessions as opposed to a single session in a randomized, sham-controlled setting, as a treatment for the inappropriate eating patterns associated with BED. Studies in populations carrying a formal ED diagnosis, with significant functional impairment and distress, are also needed.

### NIBS as a treatment for BN

The earliest publication of rTMS as a potential treatment for BN is a case report of a patient diagnosed with comorbid depression and BN who achieved an unexpected remission of binge and purge symptoms and depressive improvements after 10 sessions of 20 Hz rTMS over the left DLPFC (Hausmann et al., 2004; McClelland et al., 2013; Table 2). Follow-up studies involving high frequency left DLPFC rTMS have been mixed: one group found that a single session reduced the urge to eat, the number of binges 24 h post-rTMS, and salivary cortisol levels (Van den Eynde et al., 2010; Claudino et al., 2011), while another study found no difference between active- and sham-rTMS after 15 sessions of 20 Hz rTMS over the left DLPFC (Walpoth et al., 2008). A more recent study applied a single session of excitatory left DLPFC-rTMS in 8 female patients with BN, and reported reduced subjective ratings of craving post-rTMS, along with lower cerebral oxygenation in the DLPFC on near-infrared spectroscopy (Sutoh et al., 2016). These findings hint at the potential promise of DLPFC-rTMS for BN, which would be in keeping with the much more extensive literature demonstrating that this intervention enhances cognitive control in healthy subjects (Cho et al., 2010), and patient populations (Van den Eynde et al., 2010), with therapeutic effects in mechanistically related disorders such as addiction (Gorelick et al., 2014).

More recently, our group has shifted the rTMS stimulation target from the DLPFC to the DMPFC, as a potential treatment for major depression (Downar et al., 2014; Salomons et al., 2014; Bakker et al., 2015). As with first case report of DLPFC-rTMS for BN, we too found an unexpected remission of chronic treatment refractory binge and purge symptoms in an MDD patient with comorbid BN, following 20 sessions of 10 Hz DMPFC-rTMS. The onset of effect was rapid, occurring in the first week of treatment, and was maintained for 9 weeks post-treatment (Downar et al., 2012). In a follow-up, open-label series of 10 Hz DMPFC-rTMS in 28 ED patients with binge/purge behaviors, we noted ≥ 50% symptom reduction in 57%. On resting-state fMRI, we found increased resting-state functional connectivity in fronto-striatal salience network circuits (through DMPFC, anterior insula, and ventral striatum) specifically in the treatment responders but not non-responders (Dunlop J. et al., 2015), consistent with similar findings for DMPFC-rTMS in MDD and obsessive-compulsive disorder (Salomons et al., 2014; Dunlop K. et al., 2015). These findings suggest that DMPFC-rTMS may improve bulimic symptoms through an improvement of top-down cognitive control over urges, via frontostriatal circuits through salience-network nodes. Future work should include a sham-controlled arm, along with behavioral measures to better characterize the cognitive domains mediating the therapeutic effects of DMPFC-rTMS in BN.

### NIBS as a treatment for AN

To date, there are few published sham-controlled trials on tDCS and rTMS as treatments for AN (Bainbridge and Brown, 2014; McClelland et al., 2013). One preclinical study in a small sample of AN patients (*n* = 10) applied a single session of 10 Hz left DLPFC-rTMS, with patients reporting less anxiety and less feeling full and feeling fat (Table 2; Van den Eynde et al., 2013). An open-label case series in 5 AN patients applied 20 sessions of excitatory DLPFC-rTMS, reporting improvements in anxiety, feeling fat/full and urge to restrict/exercise over the course of treatment, enduring to 6 months; however, these effects had waned by 12-months post-treatment (McClelland et al., 2016). Another open-label series in 7 AN patients applied 10 sessions of anodal left DLPFC tDCS (Table 3), reporting improvements on the Eating Disorders Inventory (EDI) and the Eating Attitude Test (EAT) (Khedr et al., 2014). Although, these early publications are promising, further preliminary work in larger groups, with a longer course and sham control, must be performed to determine whether rTMS and tDCS are efficacious treatments for AN.

## Considerations for future studies

### Patient selection

In an attempt to limit heterogeneity, inclusion criteria for NIBS studies in ED patients are often based on DSM-5 diagnostic categories. However, as noted above, DSM-5 diagnoses still encompass substantial heterogeneity, and may conflate neurobiologically distinct endophenotypes. Future studies enrolling ED patients for NIBS trials should make efforts to at least characterize the underlying phenotypes within the clinical populations they are treating, and ideally should target a specific endophenotype associated with a specific neural substrate. Such studies should also measure behavioral or biological markers of this endophenotype to assess whether the target process was successfully engaged, and whether the engaged process did indeed mediate any observed symptomatic improvements.

### Intervention parameters

Several treatment parameters are important to consider when designing NIBS studies in ED. First, treatment parameters (protocol, total number of sessions, and number of sessions per day) needs to be selected, keeping in mind both patient convenience and therapeutic efficacy. In the older MDD-NIBS literature, 20–30 sessions of once daily rTMS is the standard protocol, with sessions lasting up to 45–60 min. However, such schedules are onerous for patients and limit overall clinic capacity. More recent studies have begun to explore much briefer protocols, such as 1–3 min theta-burst stimulation (Li et al., 2014), which have been reported to achieve equivalent or superior outcomes (Bakker et al., 2015). Other protocols, such as quadripulse stimulation (QPS), have been reported to achieve much more consistent effects across individuals (Huang et al., 2005; Tsutsumi et al., 2014). Still other recent MDD trials have delivered multiple sessions per day (up to five sessions a day), to complete the full course in 4–10 days rather than the usual 4–6 weeks (Holtzheimer et al., 2010; Baeken et al., 2014). Future ED rTMS trials should make use of these innovations to reduce patient burden, increase capacity or consistency, and accelerate the pace of improvement.

### Concurrent tasks or therapies

Another consideration for NIBS trials for ED is whether stimulation should be applied concurrently with psychotherapy or a specific cognitive/behavioral task, as opposed to simply during rest. This is especially the case if NIBS protocols are designed based on RDoC dimensions, and targets cortical regions based on abnormal activation on certain tasks. As discussed above, many areas, including the ACC/mPFC, DLPFC, insula, inferior frontal gyrus, and ventrolateral PFC are hyperactive to some tasks, but hypoactive in others. With stimulation during rest, it is difficult to assess or constrain the activation state of the underlying cortical target. Having the patient perform illness-specific cognitive task has now been shown to enhance (or reduce) the therapeutic effects of rTMS across several different indications. For example, reading trauma-related scripts during rTMS enhanced efficacy for PTSD (Isserles et al., 2013); undergoing rTMS in the presence of substance cues enhances efficacy in addiction (Dinur-Klein et al., 2014). Analogous approaches may be helpful in ED.

### Treatment target

A final consideration for ED-NIBS concerns the feasibility of the proposed target. Although, targets such as DLPFC, DMPFC, OFC, and TPJ have now been targeted in a variety of studies, others such as ventromedial prefrontal cortex or anterior insula may be difficult to reach without specially designed coils, and without also stimulating overlying structures. More feasibility studies are needed to assess how well that these areas can be engaged with rTMS and tDCS (Chib et al., 2013).

Another consideration during target selection is determining the appropriate stimulation intensity in the case of rTMS. For example, treatment intensity is determined by measuring the resting motor threshold of region of cortex directly posterior to stimulation site; in these cases, resting motor threshold is determined by the activation of the thumb or big toe for DLPFC and DMPFC, respectively (Schutter and van Honk, 2006; Hallett, 2007). It is therefore unclear for novel stimulatory sites what would be the most appropriate and reliable sites to determine optimal stimulation intensity. Studies using finite element modeling may also be helpful for optimizing stimulator placement and intensity (Nitsche et al., 2012).

The effects of rTMS also dramatically decrease the farther the site is from the scalp surface (Kozel et al., 2000), and so it is likely that stimulation intensity will have to be quite large for deep targets such as anterior insula or VMPFC. If this is the case, it is likely that pain tolerability will be a factor. In addition, trigeminal nerve pain, scalp pain, and headaches are common adverse effects associated with rTMS (Machii et al., 2006; Rossi and Hallett, 2009). Tolerability will need to be maintained when stimulating these novel targets, particularly in scalp regions with trigeminal innervation, such as the frontopolar, orbitofrontal, or temporopolar regions. This may be challenging for more intense rTMS protocols, although helmet-shaped “deep TMS” coil geometries may be somewhat helpful in allowing deep stimulation of these regions while maintaining tolerability (Roth et al., 2007). Certain targets (e.g., OFC, frontopolar cortex) may be more amenable to tDCS, which is relatively painless compared to rTMS. Another non-invasive technique worthy of future investigation is cutaneous non-invasive vagus nerve stimulation, which is also delivered via external electrodes. Its more invasive counterpart, surgically-implanted vagus nerve stimulation has recently shown some efficacy for medication-resistant depression (Ben-Menachem et al., 2015; Grimonprez et al., 2015).

Finally, stimulating multiple targets in a single session might be the optimal way to address all the abnormal behavioral dimensions in a given ED patient. Different ED symptom dimensions map to different cortical targets, and so confining stimulation to a single target may be insufficient to address multi-dimensional ED pathology. For example, in BN, excitatory stimulation of the DMPFC/insula combined with inhibitory stimulation of the VMPFC may be a more optimal strategy for enhancing cognitive control while reducing urge intensity. “Deep TMS” coils have been designed to stimulate multiple targets simultaneously (Dinur-Klein et al., 2014), and multi-channel coils allow different protocols at different targets simultaneously (Roth et al., 2014). However, the therapeutic effects of sequential vs. simultaneous stimulation have not yet been compared directly. Further research should be done to describe the safety, tolerability, clinical efficacy, and neural mechanisms of stimulating multiple targets, either sequentially or simultaneously.

## Conclusion

Neuroimaging, psychometric, and behavioral findings are converging upon a new approach to classifying psychiatric disorders, including EDs, in terms of endophenotypes or symptom dimensions. New proposed frameworks, such as the RDoC, seek to describe EDs in terms of dysfunction in specific underlying brain functions such as cognitive control, positive and negative valence, and social/self-related cognition. These functions in turn are gradually being linked to specific neurobiological processes, described at multiple levels spanning clinical symptomatology, behavioral task performance, neuroimaging studies of macro-scale network function, and cellular, molecular, and genetic mechanisms. With the advent of anatomically focal NIBS interventions, a “neuroanatomical formulation” of ED pathology may become relevant not only for basic science, but for clinical care.

At present, neuroanatomical, endophenotypic, and RDoC formulations of ED pathology must be considered tentative and preliminary. However, from available literature, it does appear that some of the tremendous and dynamic heterogeneity of symptoms in the ED population can be understood parsimoniously in terms of dysfunction in a few key cognitive systems and their associated neural circuits. For example, in BN and BED, binge and purge behaviors may acquire pathologically strong incentive salience by mechanisms similar to addiction; impaired cognitive control in turn renders binge/purge urges hard to resist, particularly during negative affect. NIBS strategies designed for addiction (e.g., enhancing cognitive control via salience-network stimulation and damping urge intensity via ventromedial stimulation) may be helpful in this setting. In ANR, this strategy may be less helpful; instead, targeting pathologically overactive negative-valence systems may address the excessive valuation of secondary over primary rewards, and the underlying compulsivity. NIBS strategies developed for OCD (such as inhibitory stimulation of the OFC and DMPFC) may be more helpful in this setting. Ancillary NIBS strategies for AN may also target distortions of body image, alexithymia, and deficits of interoception via insular, TPJ, and EBA stimulation. However, it must be acknowledged that nearly all of these approaches are at present theoretically based, and lacking even in preclinical support. The field is urgently in need of future studies in clinical populations, with adequate sample sizes and sham controls, and using endophenotypic markers to validate or refute the proposed mechanisms of action for NIBS in EDs.

To conclude, patients with EDs stand to benefit tremendously from ongoing progress in three areas: symptom characterization, diagnostic formulation, and targeted intervention. Recent initiatives will allow us to make better sense of the heterogeneity of ED pathology, both across individuals and within individuals over time. As we improve our abilities to identify robust symptom clusters, link those clusters to neural substrates, and target those substrates with NIBS interventions, treatment outcomes will improve. These advances need not occur at the expense of existing and well-validated treatment strategies involving medications, psychotherapy, and behavior modification. Rather, they will likely work in a synergistic fashion to complement and facilitate our existing treatment strategies: enhancing the cognitive control that is a prerequisitive for successful cognitive-behavioral treatments in BN, or reducing the compulsivity and rigidity that hampers behavior modification in AN. Given the considerable patient burden and chronicity of EDs, these advances in treatment options will be a welcome change for patients, families and clinicians alike.

## Author contributions

All authors listed, have made substantial, direct and intellectual contribution to the work, and approved it for publication.

## Funding

JD has received research support from the Canadian Institutes of Health Research, the National Institutes of Health, the Klarman Family Foundation, the Edgestone Family Foundation, the Toronto General and Western Hospital Foundation, and in-kind support from MagVenture for investigator-initiated studies. BW has received research support from the Ontario Mental Health Foundation, the Price Foundation, The Klarman Foundation, the Canadian Institute for Health Research, and the National Institute for Mental Health. KD has received research support from the Canadian Institutes of Health Research, Vanier Canada Scholarship Program.

### Conflict of interest statement

The authors declare that the research was conducted in the absence of any commercial or financial relationships that could be construed as a potential conflict of interest.
